# Balance improvement and reduction of likelihood of falls in older women after Cawthorne and Cooksey exercises

**DOI:** 10.1016/S1808-8694(15)31283-0

**Published:** 2015-10-20

**Authors:** Angela dos Santos Bersot Ribeiro, João Santos Pereira

**Affiliations:** 1 Physical Therapist, Master in Sciences of Human Motricity, UCB, Professor, Course of Physical Therapy, Centro Universitário de Barra Mansa; 2 Ph.D. in Medicine, UNIFESP, Full Professor, PROCIMH – UCB Universidade Castelo Branco (UCB), Rio de Janeiro, Brazil

**Keywords:** motor system, vestibular stimulation, balance, aging

## Abstract

Vestibular system is the absolute referential for the maintenance of balance. Functional deficit with aging can result in balance disturbance and in increase of likelihood of falls.

**Aim:**

To verify whether specific therapeutic approach of the system can promote motor learning and can contribute to the improvement of balance and to decrease of likelihood of falls.

**Study design:**

Clinical prospective.

**Material and Method:**

Fifteen women, aged 60 to 69, mean = 64.8 years old (±2.95), resident in Barra Mansa-RJ, were submitted to Cawthorne and Cooksey exercises during three months, three times a week, during sixty minutes. They were evaluated with Berg Balance Scale (BBS), whose scores determine the possibility of fall (PQ).

**Results:**

Comparing the data obtained before and after intervention, we observed significant difference (p < 0.05), showing improvement in BBS scores and decrease in PF.

**Conclusion:**

Cawthorne and Cooksey exercises were able to promote significant improvement in the balance of this sample and they can be applied as prevention and treatment in balance disturbances in elderly people.

## INTRODUCTION

Horak et al.^1^ considered balance as a nervous system skill to detect any instability either in advance and immediately and to generate coordinated responses that could restore the supporting base of body mass core, preventing falls. Effective maintenance of balance involves a number of central nervous system (CNS) and peripheral nervous system (PNS) structures^2^. According to Woollacott^3^, vestibular system is one of the main structures to maintain balance, given that it is considered as an absolute reference in relation to the others that also participate in this function, such as visual and somatosensorial systems.

When the set of visual, labyrinthic and proprioceptive information is not properly integrated by the CNS, there is disturbance in balance status, which can be manifested by body imbalance, and may lead to falls^4^. Aging may be responsible for these disorders. The elderly may have difficulty to precisely regulate these stimuli, which can be improved through specific training programs. Considering that daily activities are present in innumerous conditions that involve sensorial redundancy, skills to analyze and select information are essential to prevent falls^5^.

Systems in our body have physiological reserves that are characterized in the nervous system by their capability to reorganization, known as neuroplasticity^6^, ^7^. As a result of aging, reserves are reduced, but not depleted, therefore, the creation of an ideal environment for motor learning may determine a significant improvement of the function^8^. Pohl and Winstein^9^ stated that practice improves neural processing skills in the elderly as well.

Vestibular exercises, such as the ones described by Cawthorne and Cooksey, may serve as support for new arrangements of peripheral sensorial information, allowing new vestibular stimulation patterns necessary for new experiences to become automatic. This practice of balance would be capable of promoting improvement in reactions of balance and, consequently, reduce falls^10^. These exercises are part of a vestibular rehabilitation program and involve head, neck and eye movements, posture control exercises in different positions (seated, in two-leg and one-leg positions, walking), use of soft surface to reduce proprioceptive input, and exercises with closed eyes to exclude visual cues.

The purpose of the present study was to check a specific therapeutic approach for the vestibular system by the application of Cawthorne and Cooksey exercises, to see if they generated motor learning and contributed to improving balance and reducing the possibility of falls in the elderly.

## MATERIAL AND METHOD

The research project was approved by the Research Ethics Committee, Universidade Castelo Branco, according to Resolution 196, 10/10/1996, National Health Council.

### Population and sample

Elderly female patients, members of a district association in Barra Mansa/RJ, aged 60 to 69 years, were randomly chosen. All women aged 60 to 69 enrolled in the association were invited to be part of the study. The control group and the studied group were formed in a first come basis, and the 15 first applicants formed the studied group, whereas the other 15 formed the control group. They signed the informed consent term and agreed to join the study. The study was conducted between March and June 2003.

Female subjects were chosen because falls are more prevalent in women^4^, ^11^. The age range represented the first decade of aging, when responses to therapeutic interventions and physical activities are more marked.

Exclusion criteria were presence of neurological, ENT, vascular, metabolic, degenerative or neoplastic disorders, which are confirmedly known to cause balance disorders.

### Measurement instrument

Balance was assessed using Berg Balance Scale (BBS) (Annex A). This instrument is used to assess balance and risk of falls in elderly and takes into account the effect of environment. According to Gill et al.^12^, most of the falls in the elderly occur in everyday situations, especially in unfavorable environmental conditions. This scale uses 14 tests to assess subjects' skill to sit down, stand up, reach, turn around them, look over the shoulders, stand on one foot, and go upstairs. Total score is 56 and any rate equal or below 36 is associated with 100% risk of falls^13^, ^14^.

**ANNEX A:** Berg Balance Scale

1. Seated to standing up.				
Instructions: Stand up. Try not to use your hands to support you.				
Score: Check the category that applies.				
(4) able to stand up, does not use hands and stability is independent	(3) able to stand up independently, using the hands	(2) able to stand up using the hands and after many attempts	(1) needs minimum help to stand up or stabilize	(0) needs moderate or maximum help to stand up
2. Stand up with no supportInstructions: Stand for 2 minutes, without support.				
Score: Check the category that applies.				
(4) able to confidently stand for 2 minutes supervision	(3) able to stand for 2 minutes with support	(2) able to stand for 30 seconds without without support	(1) needs many attempts to stand for 30 seconds	(0) unable to stand for 30 seconds without help
IF THE SUBJECT CAN SAFELY STAND FOR 2 MINUTES, CHECK (4) IN THE ITEM SEATED WITHOUT SUPPORT AND SKIP TO CHANGING POSITION FROM STANDING UP TO SEATED.				
3. Seated without support, feet on the floor.				
Instructions: Sit down and cross your arms for 2 minutes.				
Score: Check the category that applies.				
(4) able to safely sit down for 2 minutes supervision	(3) able to sit down for 2 minutes with	(2) able to sit down for 30 seconds	(1) able to sit down for 10 seconds 10 seconds	(0) unable to sit down without support for
4. Standing up to seated positionInstructions: Sit downScore: Check the category that applies.				
(4) sits down confidently. using very little hand support	(3) controls sitting down movement using the hands	(2) uses posterior portion of the legs against the chair to control sitting down	(1) sits down independently, but controlling sitting down movement	(0) needs help to sit down
5. Transfers				
Instructions: Walk from the chair to the bed and back again. Sit once on the chair with arms and another time on the chair without arms.				
Score: Check the category that applies.				
(4) manages to safely transfer with minimum hand support	(3) manages to safely transfer with evident hand support	(2) manages to transfer with verbal cues and/or supervision	(1) needs someone to help	(0) needs two people to help or supervision to feel safe
6. Stand up without support and eyes closedInstructions: Close your eyes and stand up without moving for 10 seconds.				
Score: Check the category that applies.				
(4) manages to confidently stand for 10 seconds	(3) manages to stand for 10 seconds with supervision	(2) manages to stand for 3 seconds	(1) unable to keep eyes closed for 3 seconds, but is stable	(0) needs help not to fall
7. Stand up without help and bring feet together				
Instructions: Bring your feet together and stand without support.				
Score: Check the category that applies.				
(4) able to bring feet together independently and confidently stand for 1 minute	(3) able to bring feet together independently and stand for 1 minute with supervision	(2) able to bring feet together independently, but unable to maintain the position for 30 seconds	(1) needs help to reach the position, but manages to stand for 15 seconds	(0) needs help to reach the position and is unable to maintain it for 15 seconds – feet together
THE ITEMS THAT FOLLOW SHOULD BE CONDUCTED WHEN THE PATIENT IS STANDING WITHOUT SUPPORT.				
8. Bend forward, arms stretched				
Instructions: Bring your arms to 90º. Stretch fingers and bend your body forward as far as possible. (The examiner places a ruler on the tip of the patient's fingers when arms are at 90º. They should not touch the ruler when the patient bends forward. The recorded measurement is the distance the fingers reach when the patient is at the maximum inclination position).				
Score: Check the category that applies.				
(4) manages to confidently reach over 25 cm	(3) manages to confidently reach over 10 cm	(2) manages to confidently reach over 5 cm	(1) bends forward but needs supervision	(0) needs help not to fall
9. Take an object on the floorInstructions: Take the shoe/slipper placed in front of your feet.				
Score: Check the category that applies.				
(4) manages to confidently and easily get the slipper	(3) manages to get the slipper, but needs supervision balance independently	(2) unable to get but reaches 2.5 or 5 cm from the slipper and maintains	(1) unable to get and needs supervision while trying	(0) unable to try/needs help not to fall
10. Look back/over the right and left shoulders				
Instructions: Look backwards/over your left shoulder. Repeat over the right shoulder.				
Score: Check the category that applies.				
(4) looks back, to both sides and transfers well body weight	(3) looks only to one side, shows less weight displacement to look to the other side	(2) looks to the sides but does not manage to maintain balance	(1) needs help when turning the head	(0) needs help not to fall
11. Turn 360^o^Instructions: Turn around completely. Make a pause. Turn around completely to the other direction.				
Score: Check the category that applies.				
(4) manages to confidently turn 360° in less than 4 seconds to both sides	(3) manages to confidently turn 360° to one side in less than 4 seconds	(2) manages to confidently turn 360°, but slowly	(1) needs close supervision or verbal cues	(0) needs help while turning
DYNAMIC DISPLACEMENT OF WEIGHT WHILE STANDING WITHOUT SUPPORT.				
12. Count the number of times you step on a bench				
Instructions: Place each foot alternatively over the bench. Keep on doing it until each of them have touched it 4 times.				
Score: Check the category that applies.				
(4) able to stand up independently and confidently, and takes 8 steps within 20 seconds	(3) able to stand up independently and takes 8 steps in over 20 seconds	(2) manages to take 4 steps without help, with supervision	(1) manages to take more than 2 steps, but needs minimum help	(0) needs help not to fall/unable to try
13. Stand without support one foot in front of the other				
Instructions: (Show the subject). Place one foot in front of the other. If you do not manage to have one in front of the other, take the longest step you can to have your toes touch your ankle.				
Score: Check the category that applies.				
(4) (3) manages to place the feet correctly and independently and maintains the position for 30 seconds 30 seconds	(2) manages to place the feet correctly and independently and maintains the position for	(1) manages to take a small step independently and maintains the position for 30 seconds seconds.	(0) needs help to take the step, but is able to maintain the position 15	loses balance when takes a step forward or stands up
14. Stand up on one foot				
Instructions: Stand up on one foot as long as you can, but without support.				
Score: Check the category that applies.				
(4) manages to raise the leg independently and maintains the position for over 10 seconds	(3) manages to raise the leg independently and maintains the position for 5-10 seconds	(2) manages to raise the leg independently and maintains the position for over 3 seconds	(1) tries to raise the leg; unable to maintain the position for 3 seconds, but remains standing up independently	(0) does not manage to try or needs help not to fall
TOTAL SCORE _____				
MAXIMUM SCORE _____				

This instrument shows excellent reliability (0.96) and moderate to high correlation with other balance functional assessment instruments, such as Barthel Mobility Scale, 0.67; Up and Go Test, 0.76; Tinetti Balance Scale, 0.91^15^. The scale has excellent test-retest objectivity (ICC = 0.98)^16^.

Absolute scores obtained in BBS were applied to reach the rate of Likelihood of Fall (PQ) using the following equation: 100% × exp (10.46 – 0.25 × BBS score + 2.32 × history of instability)/[1 + exp (10.46 – 0.25 × BBS score + 2.32 × history of instability)], and BBS score is the score obtained by the subject in the BBS. History of instability receives value 0 if there is no report of instability and value 1, if there is report of instability.

### Procedures

Out of forty-five subjects that responded the invitation, 33.3% (15 subjects) did not participate in the study because they did not comply with the inclusion criteria or were not interested. The percentages were: 46.6% (7 subjects) presented metabolic disorders (diabetes mellitus); 6.6% (1 subject), vascular disorder (uncontrolled high blood pressure); 13.3% (2 subjects) had neurological affections (sequelae of cerebral vascular accident); 40% (6 subjects) had ENT affections (labyrinthitis); 13.3% (2 subjects) had significant visual deficit, and 13.3% (2 subjects) were able to participate but did not show interest. The sum up of percentages is higher than 100% because subjects could have manifested more than one disorder. Thus, we selected 30 subjects that participated in the study.

They were all assessed by BBS and after nine weeks, they were reassessed. Components of the studied group were submitted to vestibular stimulation with Cawthorne and Cooksey exercises, three times a week, for sixty minutes during this time interval (Annex B). The interval represented the mean time recommended to assess the progression of a patient submitted to vestibular rehabilitation^17^.

**ANNEX B:** Cawthorne and Cooksey Exercises

A) Eye and head movement, sitting down - first slowly, then faster: 1) Look up and down; 2) Look to the right and to the left; 3) Bring your fingers closer and farther, looking at it; 4) Move your head (slowly and then faster) to the right and to the left, with open eyes; 5) Move your head (slowly and then faster) up and down, with open eyes; 6) Repeat 4 and 5 with closed eyes. B) Head and body movement, sitting down: 1) Place an object on the floor. Take it and bring it above your head and place it on the floor again (look at the object the whole time); 2) Shrink your shoulders and make circular movements; 3) Bend forward and take an object through the back and front of your knees. C) Standing up exercises: 1) Repeat A and B2; 2) Sit down and stand up, sit down and stand up again; 1) Sit down and stand up; Sit down and stand up again with closed eyes; 2) Stand up, but turn to the right while standing; 3) Stand up, but turn to the left while standing; 4) Throw a small ball from one hand to the other (above the horizon level); 5) Throw a small ball from one hand to the other under your knees and alternatively.
Other activities to improve balance: 1) Climb up and downstairs (use handrail, if necessary); 2) Stand up and take sudden 90o turns (first with open eyes, then with closed eyes); 3) While walking, look to the right and to the left (as if you were reading labels in the market; 4) Practice standing on one foot (with the right foot then the left foot), first with open eyes, then with closed eyes; 5) Stand up, on a soft surface; a) Walk on the surface to get used to it; b) Walk on the tip of your feet first with open eyes, then with closed eyes; c) Practice exercise 4 on a soft surface; 6) Circle around a person that is on the center that throws a large ball (which should be thrown back); 7) Walk around the room with closed eyes.

Barbosa MSM et al. Reabilitaçã o Labiríntica: o que ée como se faz. Rev Bras Med Otorrinolaringol 1995; 2(1): 24-34

All participants presented a percentage of attendance to the program sessions that was equal or higher than 75%, so that data could be included in the analysis.

### Data analysis

Initial and final data were analyzed and compared by two statistical tests of significance: T-Student test and Wilcoxon test. T-Student test requires variables to be distributed as regular likelihood. To check this assumption we used Kolmogorov-Smirnov test. Level of significance (α) was 5%, that is, p < 0.05^18^.

## RESULTS AND DISCUSSION

Mean age of the studied group was 64.8 years (±2.95) and to the control group it was 65.46 years (±2.85).

**Graph 1:**
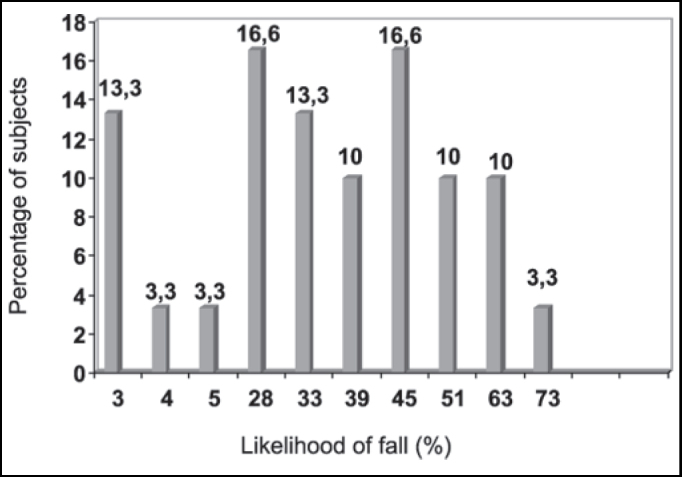
Quantity (in percentage) of subjects and their respective rate of likelihood of fall (PQ) in the 1st assessment

Subjects presented a first assessment of BBS scores with the respective PQ showed in Table 1. The percentage of subjects in each PQ value is shown in Graph 1. No elderly subject presented PQ equal to 100%, which would be expectable in a survey with healthy elderly, but 24 of them (79.8%) presented PQ between 28% and 73%, which demonstrates that subjects without diseases present likelihood of suffering falls that is high enough to restrict their daily life activities. The process of aging, per se, determines gradual system failure, regardless of the presence of disorders, but there was a minimum of general BBS score, which inevitably occurs during the process, that is significant considering non-linear progression. Shumway-Cook et al.^15^ reported that 25% to 35% of the population aged over 65 years tends to suffer falls.

**Table 1:** Berg Balance Scale score and respective rate of Likelihood of fall in the 1st assessment.

Number of subjects	n = 4	n = 1	n = 1	n = 5	n = 4	n = 3	n = 5	n = 3	n = 3	n = 1
BBS score/report of instability	56/0	55/0	54/0	55/1	54/1	53/1	52/1	51/1	49/1	47/1
Likelihood of fall	3%	4%	5%	28%	33%	39%	45%	51%	63%	73%

**Graph 2:**
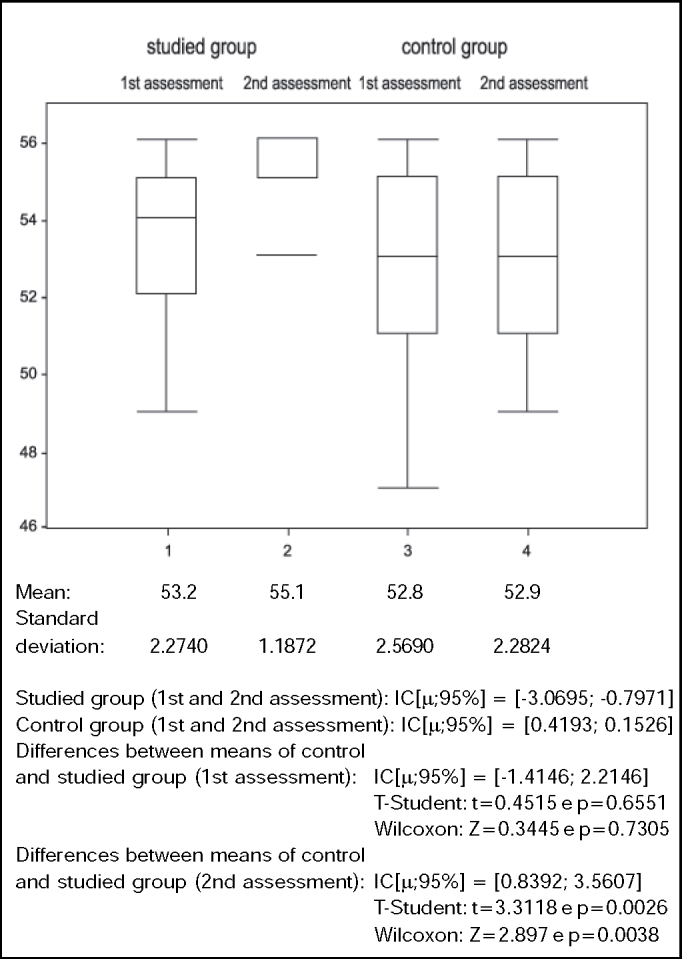
Values of Berg Balance Scale (score) in control and studied groups in 1st and 2nd assessment

**Graph 3:**
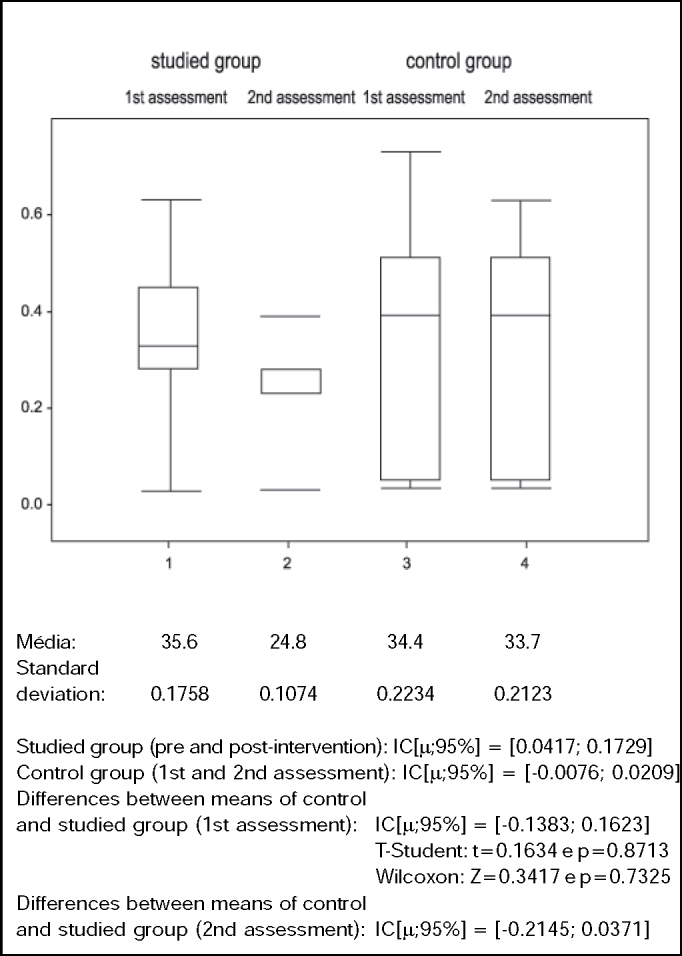
Values of Likelihood of fall (%) in control and studied groups in the 1st and 2nd assessment.

Data presented in Graphs 2 and 3 detected homogeneity between both groups in the 1st assessment, because there were no statistically significant differences in results (p > 0.05).

This study managed to demonstrate that BBS is sensitive to detecting abnormalities in balance of healthy elderly. The elderly in the studied and control groups did not present statistically significant difference when assessed for the first time (p > 0.05), but for the second assessment we observed statistically significant differences between the two groups (p < 0.005), provided by significant improvement in balance (p < 0.05) in the studied group after intervention (Table 2 and Graph 2). There was significant improvement in PQ (p < 0.05) for the studied group, with reduction of 30.4% in the likelihood of having falls (Table 3 and Graph 3), leading to the conclusion that a specific intervention, based on stimulation of specific system, generated positive functional responses in this studied group. The clinical importance of this result lies in the fact that falls are one of the main factors that contribute to morbidity and mortality of the elderly. Thus, preventing falls by improving balance provides basic conditions for the maintenance of physical independence.

**Table 2:** Berg Balance Scale by subject in the studied group in the 1st and 2nd assessments (pre and post- intervention).

Subjects	1	2	3	4	5	6	7	8	9	10	11	12	13	14	15
1st assess.	55	52	52	56	51	55	54	54	56	54	55	49	52	49	54
2nd assess.	56	53	56	56	56	56	55	55	56	55	56	56	53	53	55
X1 = 53.2	dp = 2.27				T-Student t = -3.6495					Wilcoxon Z = -2.6858					
X2 = 55.1	dp = 1.18				(values) p = 0.0026					(values) p = 0.0072

**Table 3:** Values of likelihood of fall by subjects in the studied group in the 1st and 2nd assessment (pre and post-intervention).

Subjects	1	2	3	4	5	6	7	8	9	10	11	12	13	14	15
1st assess.	28%	45%	45%	3%	51%	28%	33%	33%	3%	33%	28%	63%	45%	63%	33%
2nd assess.	23%	39%	23%	3%	23%	23%	28%	28%	3%	28%	23%	23%	39%	39%	28%
X1 = 35.6%	dp = 0.1758				T-Student t = 3.5107					Wicoxon Z = 2.3965					
X2 = 24.8%	dp = 0.1074				(values) p = 0.0035					(values) p = 0.0166					

Differences between the control and studied group to PQ both in the first and second assessment were not significant (p > 0.5 and p > 0.05, respectively), despite the tendency towards increase in difference between both groups in the 2nd assessment, which means that significant improvement obtained in the studied group, confirmed in this study, may not be extrapolated to other groups (Graph 3).

Data in Tables 4 and 5 and in Graphs 2 and 3 show, respectively, that the control group did not present significant differences between means of BBS (p > 0.05) in the 1st and 2nd assessments nor between PQ means (p > 0.05).

**Table 4:** Berg Balance Scale score by subject in the control group in the 1st and 2nd assessment

Subjects	1	2	3	4	5	6	7	8	9	10	11	12	13	14	15
1st assess.	51	53	53	55	47	52	55	54	56	56	55	53	51	52	49
2nd assess.	51	53	53	55	49	52	55	54	56	56	55	53	51	52	49
X1 = 52.8	dp = 2.56				T-Student t = -1					Wilcoxon Z = -0.021					
X2 = 52.9	dp = 2.28				(values) p = 0.3343					(values) p = 0.9833

**Table 5:** Values da Likelihood of fall by subjects in the control group in the 1st and 2nd assessment

Subjects	1	2	3	4	5	6	7	8	9	10	11	12	13	14	15
1st assess.	51%	39%	39%	28%	73%	45%	4%	5%	3%	3%	28%	39%	51%	45%	63%
2nd assess.	51%	39%	39%	28%	63%	45%	4%	5%	3%	3%	28%	39%	51%	45%	63%
X1 = 34.4%	dp = 0.223				T-Student t = 1					Wilcoxon Z = 0.0209					
X2 = 33.7%	dp = 0.212				(values) p = 0.3343					(values) p = 0.9833					

## CONCLUSIONS

The results found in this study confirmed that according to BBS, healthy elderly subjects have balance disorders and run the risk of falling.

Cawthorne and Cooksey exercises applied as described in the procedures were capable of improving balance in our sample, consequently reducing the likelihood of fall.

The results and conclusions described here confirmed the expectations of different authors^19^, ^20^, ^21^, ^22^ that suggested improvement in balance and in likelihood of falls when there was application of vestibular stimulation in healthy elderly, given that these subjects have really presented significant improvement.

Elderly subjects, who reported or not presence of posture instability and/or the event of fall, should be submitted to vestibular stimulation exercises, exercises that are easy to apply and affordable, which are preventive and curative concerning balance deficits and risk of falls. Considering that falls are aspects that substantially change the quality of life of the elderly and that life expectancy of the population in general has increased significantly, leading to increasingly higher elderly population every year, general therapeutic interventions directed to the elderly and specially those that provide prevention of falls owing to improvement of posture stability, will eventually lead to improvement in quality of life of this part of the population, which is currently the priority of any and all health policies.
